# Static and dynamic functional connectivity combined with the triple network model in amnestic mild cognitive impairment and Alzheimer's disease

**DOI:** 10.3389/fneur.2023.1284227

**Published:** 2023-11-17

**Authors:** Qi Feng, Luoyu Wang, Xue Tang, Hanjun Hu, Xiuhong Ge, Zhengluan Liao, Zhongxiang Ding

**Affiliations:** ^1^Department of Radiology, Hangzhou First People's Hospital, Hangzhou, China; ^2^School of Medical Imaging, Hangzhou Medical College, Hangzhou, China; ^3^Fourth Clinical School, Zhejiang Chinese Medical University, Hangzhou, China; ^4^Department of Psychiatry, Zhejiang Provincial People's Hospital/People's Hospital of Hangzhou Medical College, Hangzhou, China

**Keywords:** Alzheimer's disease, amnestic mild cognitive impairment, functional connectivity, dynamic FC, default-mode network

## Abstract

**Background:**

Alzheimer's disease (AD) and amnestic mild cognitive impairment (aMCI) are characterized by abnormal functional connectivity (FC) of default-mode network (DMN), salience network (SN), and central executive network (CEN). Static FC (sFC) and dynamic FC (dFC) combined with triple network model can better study the dynamic and static changes of brain networks, and improve its potential diagnostic value in the diagnosis of AD spectrum disorders.

**Methods:**

Differences in sFC values and dFC variability patterns among the three brain networks of the three groups (53 AD patients, 40 aMCI patients, and 40 NCs) were computed by ANOVA using Gaussian Random Field theory (GRF) correction. The correlation between FC values (sFC values and dFC variability) in the three networks and cognitive scores (MMSE and MoCA) in AD and aMCI groups was analyzed separately.

**Results:**

Within the DMN network, there were significant differences of sFC values in right/left medial superior frontal gyrus and dFC variability in left opercular part inferior frontal gyrus and right dorsolateral superior frontal gyrus among the three groups. Within the CEN network, there were significant differences of sFC values in left superior parietal gyrus. Within the SN network, there were significant differences of dFC variability in right Cerebelum_7b and left opercular part inferior frontal gyrus. In addition, there was a significant negative correlation between FC values (sFC values of CEN and dFC variability of SN) and MMSE and MoCA scores.

**Conclusion:**

It suggests that sFC, dFC combined with triple network model can be considered as potential biomarkers for AD and aMCI.

## Introduction

The onset of Alzheimer's disease (AD) causes cognitive decline, personality changes and behavioral and psychological symptoms, seriously affects the quality of life, significantly increases all-cause mortality, and is an important factor for mortality and disability in the elderly, which has caused a huge economic burden. Amnestic mild cognitive impairment (aMCI) individuals present with memory decline and cognitive decline in elderly who do not meet the criteria for AD. It is considered to be the prodromal stage of AD. Resting-state functional magnetic resonance imaging (rs-fMRI) is a non-invasive method and do not use radioactive contrast agents. Rs-fMRI uses blood oxygen level-dependent (BOLD), and the difference of susceptibility between arterial blood and venous blood can be used as an intrinsic contrast agent under certain conditions. The impairment of neuronal function caused by AD can disconnect brain functional areas. Therefore, brain functional connectivity (FC) research has become an important method to observe the changes of brain function in AD.

In 2011, Menon proposed the concept of “triple network model” including default-mode network (DMN), salience network (SN), and central executive network (CEN) (1). The three networks closely interact to regulate human cognitive and affective states. The resting-state networks composed of PCC, medial prefrontal cortex and inferior parietal lobule was called DMN (2). The DMN is the most active network when the brain is not in a task, which can monitor the changes of the internal environment, automatically collect information from the external environment, process and store it (3). The ECN, which includes the posterior parietal cortex and dorsolateral prefrontal cortex, is involved in episodic memory retrieval and psychological processes of self-reference and plays a role in decision making in goal oriented behavior (4). In addition, the SN is composed of the frontal insular cortex and the dorsal anterior cingulate cortex, in the various internal and external stimulation to identify the most relevant stimulus to guide behavior (5, 6). In most of the fMRI studies, the DMN has attracted the most attention because we can observe changes in its FC in AD, MCI, and high-risk AD subjects (7, 8). In healthy young populations, the SN has been reported to drive the DMN and CEN in both resting and task states (9). Zhou et al. reported increased FC within the SN in AD group (10). Recent studies have revealed changes in directional FC within and among the three brain networks in AD and MCI (11, 12). Further studies on the alterations of the triad network pattern in AD and aMCI will help us to better understand their brain network pathological mechanisms.

In the seed-based method, the researcher selects a region of the brain of interest and then extracts the activation time course. This time course was then tested for correlation with the time course of other brain voxels (13). Those regions that showed a high positive correlation with seed points were considered functionally coupled. There are many seed-based methods for studying the FC in AD and MCI patients (5, 14–17). After a large number of studies, analysis of rs-fMRI data based on independent component analysis (ICA) has been used to identify intrinsic network connectivity well (18–21). Both the seed and ICA methods tended to reveal the same networks. ICA can detect multiple brain networks simultaneously, but separating noise-related components and determining the optimal number of components is not standard (22).

Recent studies have found that resting state FC has periodic changes in strength and direction. Chang and Glover showed that the correlation and FC timing of the posterior cingulate gyrus and other DMN nodes would change with time (23). Previous rs-fMRI studies showed that brain FC can change transiently within a short time window, which called dynamic FC (dFC) (24). Dynamic FC has become an important indicator in rs-fMRI studies by capturing time fluctuations in brain FC during MR scanning (24). At present, a variety of methods can be used to study dFC, among which the sliding time window technique is the most widely used one to evaluate the correlation between points of interest or voxels under different time windows (25). Prior studies have shown that quantifying dFC patterns may be a sensitive biomarker to assess disease progression (26, 27). Wang et al. have combined static FC (sFC) and dFC to analyze the abnormalities in the anterior and posterior hippocampus of subjective cognitive decline (SCD) patients (16). A study have found that both aMCI and SCD show varying degrees of dFC variability in triple network model (17). There have been some studies using sFC or dFC to study brain networks in AD spectrum diseases (17, 28–31), but there are very few studies combining the two.

In the present study, we aimed to use rs-fMRI data to discovery the static and dynamic FC changes of DMN, SN and CEN in AD and aMCI, and to reveal the evolution rules of the sFC and dFC of the three networks in the process of AD, in order to provide strong neuroimaging evidence in the early diagnosis. In addition, the correlation between FC values (sFC values and dFC variability) in the three networks and cognitive scores (MMSE and MoCA) in AD and aMCI patients will be analyzed separately.

## Materials and methods

### Study cohort

From September 2016 to February 2020, 63 patients with AD and 45 patients with aMCI were enrolled in Zhejiang Provincial People's Hospital. A total of 44 normal controls (NC) were recruited. This study was approved by the Ethics Committee of Zhejiang Provincial People's Hospital (No. 2012KY002). All procedures in accordance with the declaration of Helsinki. All subjects were right-handed and gave written informed consent prior to the experiment. All participants performed routine brain magnetic resonance imaging (MRI), Mini-mental state examination (MMSE), and Montreal cognitive assessment scale (MoCA). The inclusion and exclusion criteria for AD, aMCI and NC were referred to our previous study (32). AD inclusion criteria: patients with AD met the revised NINCDS-ADRDA (National Institute of Neurological and Communicative Disorders and Stroke and the Alzheimer's Disease and Related Disorders Association) criteria for “probable AD” with MMSE score ≤24 and MoCA score ≤26. AMCI inclusion criteria: chief complaint memory impairment; the clinical manifestations were normal; MMSE score >24 and ≤27. Admission criteria for NC subjects: no neurological defects such as hearing or vision impairment; no stroke, epilepsy, depression or other neurological or psychiatric diseases; conventional brain MRI showed no infarction, hemorrhage, tumor lesions; MMSE score ≥28.

Some subjects with missing images or head movement (6 AD, 3 aMCI, and 3 NCs) and subjects with missing mental scale data (4 AD, 2 aMCI, and 1 NC) were excluded. Finally, 53 patients with AD, 40 patients with aMCI, and 40 NCs were enrolled.

### MRI acquisition

Data acquisition was performed using a Discovery MR750 3.0 T MR scanner with a standard head coil. In order to rule out relevant brain diseases, routine brain MRI scans were performed first. Then we collected the high-resolution three-dimensional T1-weighted magnetization-prepared rapid gradient echo (3D-T1 MPRAGE) sequence. The scanning parameters: echo time (TE) = 2.9 ms, repeat time (TR) = 6.7 ms, turnover Angle = 12°, inversion time (TI) = 450 ms, FOV = 256 × 256 mm^2^, layer thickness/layer spacing = 1/0 mm. Matrix = 256 × 256, total 192 layers of sagittal sections. Finally, rs-fMRI sequence acquired using echo plane imaging (EPI). Scanning parameters: TE = 30 ms, TR = 2,000 ms, turnover Angle = 90°, FOV = 220 × 220 mm^2^, and layer thickness/layer spacing = 3.2/0 mm. It contained 210 time points, each of which had 44 slices. The subjects were asked to remain still, not to have any thought activity and not to fall asleep during the scanning.

### Preprocessing of rs-fMRI

Rs-fMRI Data was preprocessed based on Data Processing & Analysis for Brain Imaging (DPABI) (33) based on MATLAB (Matrix Laboratory) platform including the following steps: (1) Convert DICOM to NIFTI format; (2) Delete the first 10 time points; (3) Level time correction and head motion correction; (4) Standardization to MNI standard space using 3D-T1 MPRAGE images, resampling is 3 mm × 3 mm × 3 mm; (5) Eliminate linear trends; (6) a noise removal including white matter signals, cerebrospinal fluid signals, and Friston-24 head motion parameters; and (7) bandpass filtering (0.01–0.1 Hz).

### Estimation of static and dynamic functional connectivity

We used a seed-based approach to extract DMN, SN and CEN. The definition of the three seeds in three networks was based on previous fMRI studies (5, 34). The seed of DMN was set at the posterior cingulate cortex (PCC) (χ = 0, y = −53, z = 26). The seed of SN was set in the dorsal anterior cingulate cortex (dACC) (χ = 10, y = 34, z = 24). The seed of the CEN was set in the dorsolateral prefrontal cortex (dlPFC) (χ = 30, y = 12, z = 60). The diameter of the seed point was set to 6 mm.

Static FC calculation process was as follows: The Pearson correlation coefficient between the time series of voxels within each seed point and the time series of each voxel in the whole brain was determined, reflecting the brain static connectivity pattern.

The dFC model was characterized using the sliding window method, which cuts the ROI time series into several short segments. The coefficient of variation (CV) map was computed across time windows. In order to be consistent with the previous dynamic rs-fMRI studies (35–38), we used a 50 TR sliding window length and a 2 TR step length. We have further verified our results using 60 TR sliding window length and a 2 TR step size and added the figures for validation in the Supplementary Figures 1, 2. For each sliding window, a correlation plot was generated by calculating the time correlation coefficients between the truncated time series of seed points in the three brain networks and all other voxels. Then, the CV map was calculated to quantify variability of dFC. To improve the normality of the correlation distribution, each correlation graph was converted to a *z*-valued map using Fisher's *r*-to-*z* transform. Finally, a 6 mm full width at half maximum Gaussian kernel was used for smoothing.

### Statistical analysis

The statistics of demographic and psychiatric scales were performed on SPSS 22.0 software. Analysis of variance (ANOVA) was used to compare age, education level, MMSE score, and MoCA score among AD, aMCI, and NC groups. The data of demographic variables were classified by Chi-square test. A *post hoc* test was then performed for statistically significant differences.

We have performed one sample *T* tests first in all three groups and observed typical DMN, CEN, and SN patterns. We have included the relevant figures in the Supplementary Figures 3–11. Differences in sFC values and dFC variability patterns among the triple networks of the three groups were computed by ANOVA on DPABI based on MATLAB. We regressed four covariates: age, sex, education level, and head movement. A gray matter template was applied to remove interference such as white matter and cerebrospinal fluid. The resultant F-maps were thresholded using Gaussian Random Field theory (GRF) correction with voxel *P* < 0.001 and cluster *P* < 0.05. The brain regions with significant inter-group differences were then examined *post hoc* test using SPSS software. Select Bonferroni correction to adjust for multiple comparisons (*P* < 0.01).

The correlation between FC values (sFC values and dFC variability) of the three brain networks and cognitive scores (MMSE and MoCA) in patient groups (AD and aMCI) was analyzed separately regressed out the effects of head movement, age, gender, and education. Bonferroni correction was further used, and *P* < 0.01 was considered statistically significant.

## Results

### Demographic and cognitive scale data

The demographic and cognitive scale data of all study subjects were shown in Table 1. Among the three groups, there were no significant differences in age, gender, and education level (*P* > 0.05). However, MMSE and MoCA scores were significantly different (*P* < 0.05). *Post hoc* analysis showed that the AD group had the worst performance in MMSE and MoCA scores (*P* < 0.05).

**Table 1:** Demographics performances of the three groups.

	**AD**	**aMCI**	**NC**	***p* value**	***posthoc* ^c^**
Sample size	53	40	40	NA	NA
Age (years, mean ± SD)	66.830 ± 7.849	66.225 ± 8.313	65.850 ± 9.178	0.851 ^b^	NA
Gender (Male: Female)	20: 33	23: 17	19: 21	0.166^a^	NA
Education (years, mean ± SD)	7.359 ± 4.359	7.350 ± 3.051	7.375 ± 3.378	1.000^b^	NA
MMSE	18.717 ± 4.538	26.275 ± 0.877	28.950 ± 0.904	< 0.001^b^	NC>aMCI>AD
MoCA	5.492 ± 0.754	22.650 ± 2.327	27.350 ± 1.350	< 0.001^b^	NC>aMCI>AD

a p-values for the chi-square test;

b p-value for the analysis of variance;

c *Post hoc* testing obtained by Bonferroni correction. AD, Alzheimer's disease; aMCI, amnestic mild cognitive impairment; NC, normal controls; SD, standard deviation; MMSE, mini-mental state examination; MoCA, Montreal cognitive assessment scale.

### Static FC results of the triple networks

**Figure 1:**
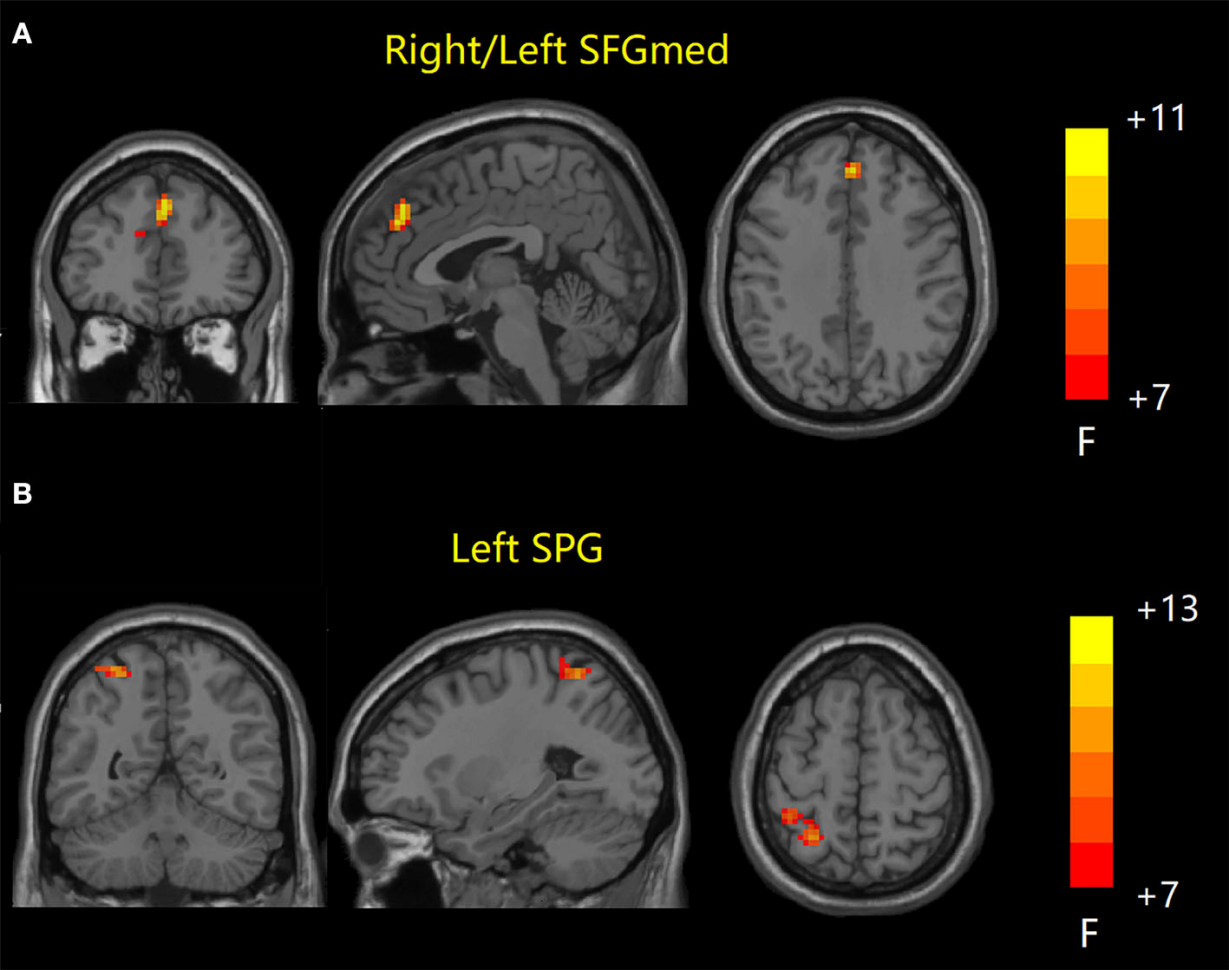
Static FC results among the three groups. **(A)** Brain regions with significant differences in sFC values in DMN network. **(B)** Brain regions with significant differences in sFC values in CEN network. FC, functional connectivity; sFC, static FC; DMN, default mode network; CEN, central executive network; SFGmed, medial superior frontal gyrus; SPG, superior parietal gyrus.

**Figure 2:**
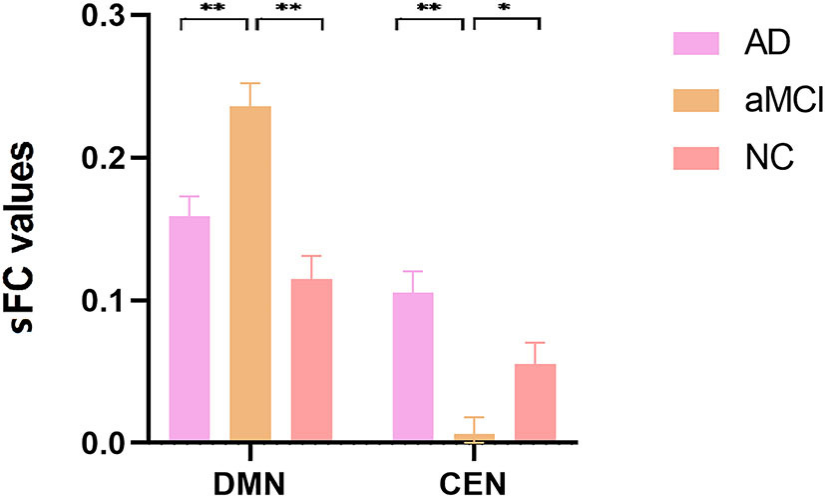
*Post hoc* comparisons of analysis of variance. The connection between two pillars represents significant between-group differences (*P < 0.05, **P < 0.01, Bonferroni correction). The error bars refer to the standard error. sFC, static functional connectivity; AD, Alzheimer's disease; aMCI, amnestic mild cognitive impairment; NC, normal controls; DMN, default mode network; CEN, central executive network.

ANOVA results across the three groups in sFC were showed in Table 2. Within the DMN network, we observed significant differences in sFC values of right/left medial superior frontal gyrus (SFGmed) among three groups (GRF correction, voxel *P* < 0.001, cluster *P* < 0.05) (Figure 1A). *Post hoc* analyses showed significant differences in sFC values between AD vs. aMCI and between aMCI vs. NC (Bonferroni correction, *P* < 0.01) (Figure 2). Within the CEN network, we observed significant differences in sFC values of left superior parietal gyrus (SPG) among three groups (GRF correction, voxel *P* < 0.001, cluster *P* < 0.05) (Figure 1B). *Post hoc* analyses showed significant differences in sFC values between AD vs. aMCI (Bonferroni correction, *P* < 0.01) and between aMCI vs. NC (Bonferroni correction, *P* < 0.05) (Figure 2). In the SN network, no brain region was found to be significantly different among the three groups.

**Table 2:** ANOVA results across the three groups in static FC in DMN and CEN.

**Brain network**	**Anatomical region**	**Number of voxels**	**Peak MNI coordinates (*x*, *y*, *z*)**	**Peak intensity**
DMN	Right/Left SFGmed	46	3, 42, 36	11.679
CEN	Left SPG	48	−27, −54, 60	11.100

DMN, default mode network; CEN, central executive network; MNI, Montreal Neurological Institute; SFGmed, medial superior frontal gyrus; SPG, superior parietal gyrus.

### Dynamic FC results of the triple networks

**Figure 3:**
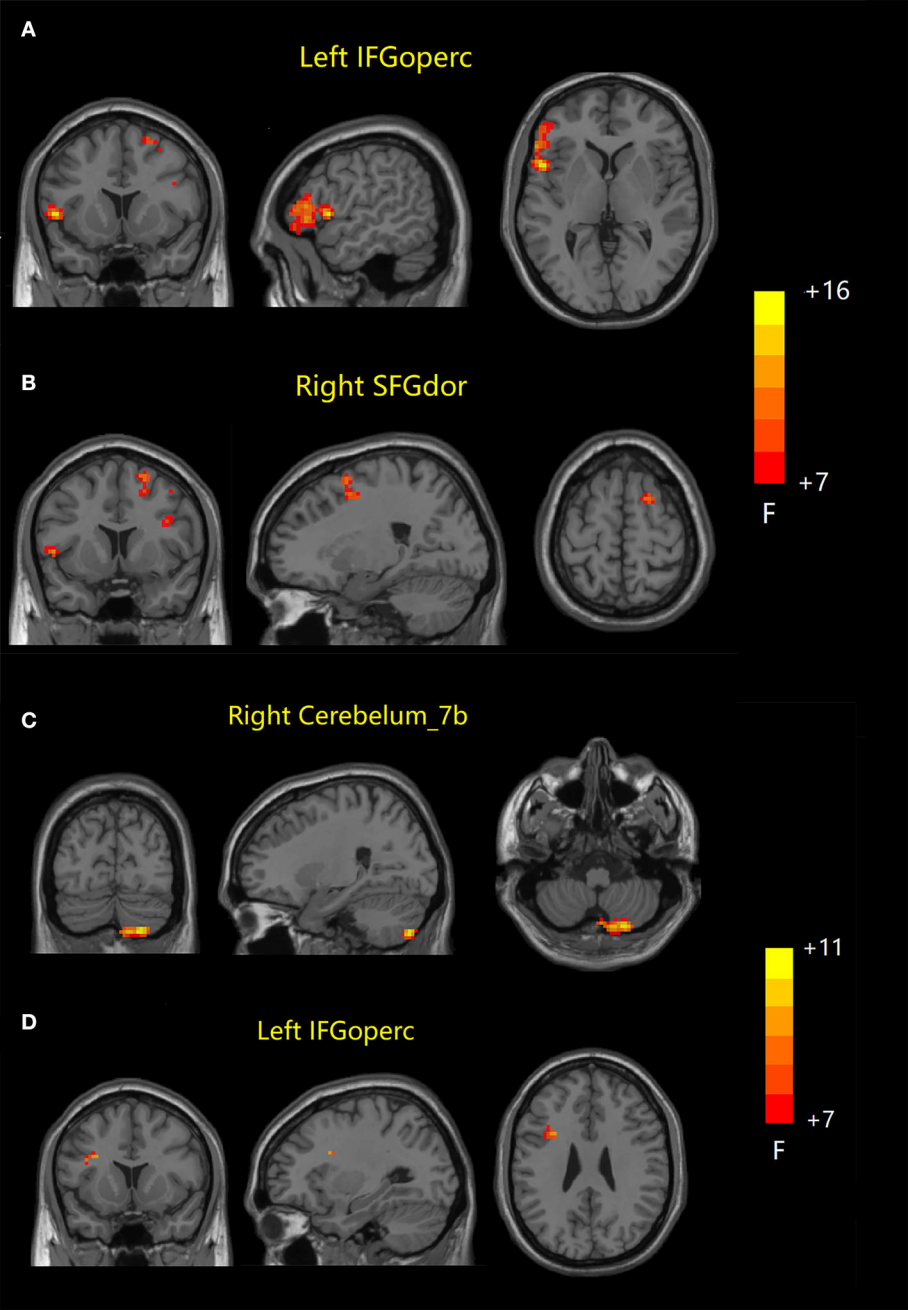
Dynamic FC results among the three groups. **(A, B)** Brain regions with significant differences in dFC variability in DMN network. **(C, D)** Brain regions with significant differences in dFC variability in SN network. FC, functional connectivity; dFC, dynamic FC; DMN, default mode network; SN, salience network; IFGoperc, opercular part inferior frontal gyrus; SFGdor, dorsolateral superior frontal gyrus; IFGoperc, opercular part inferior frontal gyrus.

**Figure 4:**
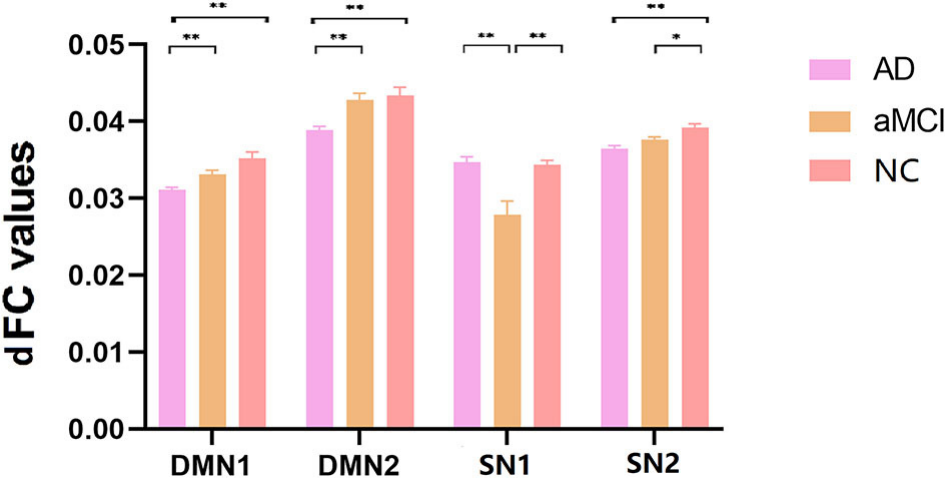
*Post hoc* comparisons of analysis of variance. The connection between two pillars represents significant between-group differences (*P < 0.05, **P < 0.01, Bonferroni correction). The error bars refer to the standard error. dFC, dynamic functional connectivity; AD, Alzheimer's disease; aMCI, amnestic mild cognitive impairment; NC, normal controls; DMN, default mode network; SN, salience network.

ANOVA results across the three groups in dFC variability were showed in Table 3. Within the DMN network, there were significant differences in dFC variability of left opercular part inferior frontal gyrus (IFGoperc) (DMN1) and right dorsolateral superior frontal gyrus (SFGdor) (DMN2) among three groups (GRF correction, voxel *P* < 0.001, cluster *P* < 0.05) (Figures 3A, B). *Post hoc* analyses showed significant differences in dFC variability between AD vs. aMCI (Bonferroni correction, *P* < 0.01) and AD vs. NC (Bonferroni correction, *P* < 0.01) (Figure 4). Within the SN network, there were significant differences in dFC variability of right Cerebelum_7b (SN1) and left opercular part inferior frontal gyrus (IFGoperc) (SN2) among the three groups (GRF correction, voxel *P* < 0.001, cluster *P* < 0.05) (Figures 3C, D). *Post hoc* analyses showed significant differences in dFC variability between AD vs. aMCI (Bonferroni correction, *P* < 0.01) and aMCI vs. NC (Bonferroni correction, *P* < 0.01) for right Cerebelum_7b, AD vs. NC (Bonferroni correction, *P* < 0.01) and aMCI vs. NC (Bonferroni correction, *P* < 0.05) for left IFGoperc (Figure 4). In the CEN network, there was no brain region found to be significantly different among the three groups.

**Table 3:** ANOVA results across the three groups in dynamic FC variability in DMN and SN.

**Brain network**	**Anatomical region**	**Number of voxels**	**Peak MNI coordinates (*x*, *y*, *z*)**	**Peak intensity**
DMN	Left IFGoperc	116	−54, 12, 3	15.037
DMN	Right SFGdor	36	21, 9, 63	11.509
SN	Right Cerebelum_7b	23	24, −78, −51	11.0924
SN	Left IFGoperc	11	−30, 12, 27	9.503

DMN, default mode network; SN, salience network; MNI, Montreal Neurological Institute; IFGoperc, opercular part inferior frontal gyrus; SFGdor, dorsolateral superior frontal gyrus; IFGoperc, opercular part inferior frontal gyrus.

### Correlation between mental cognition scales and static and dynamic FC

**Figure 5:**
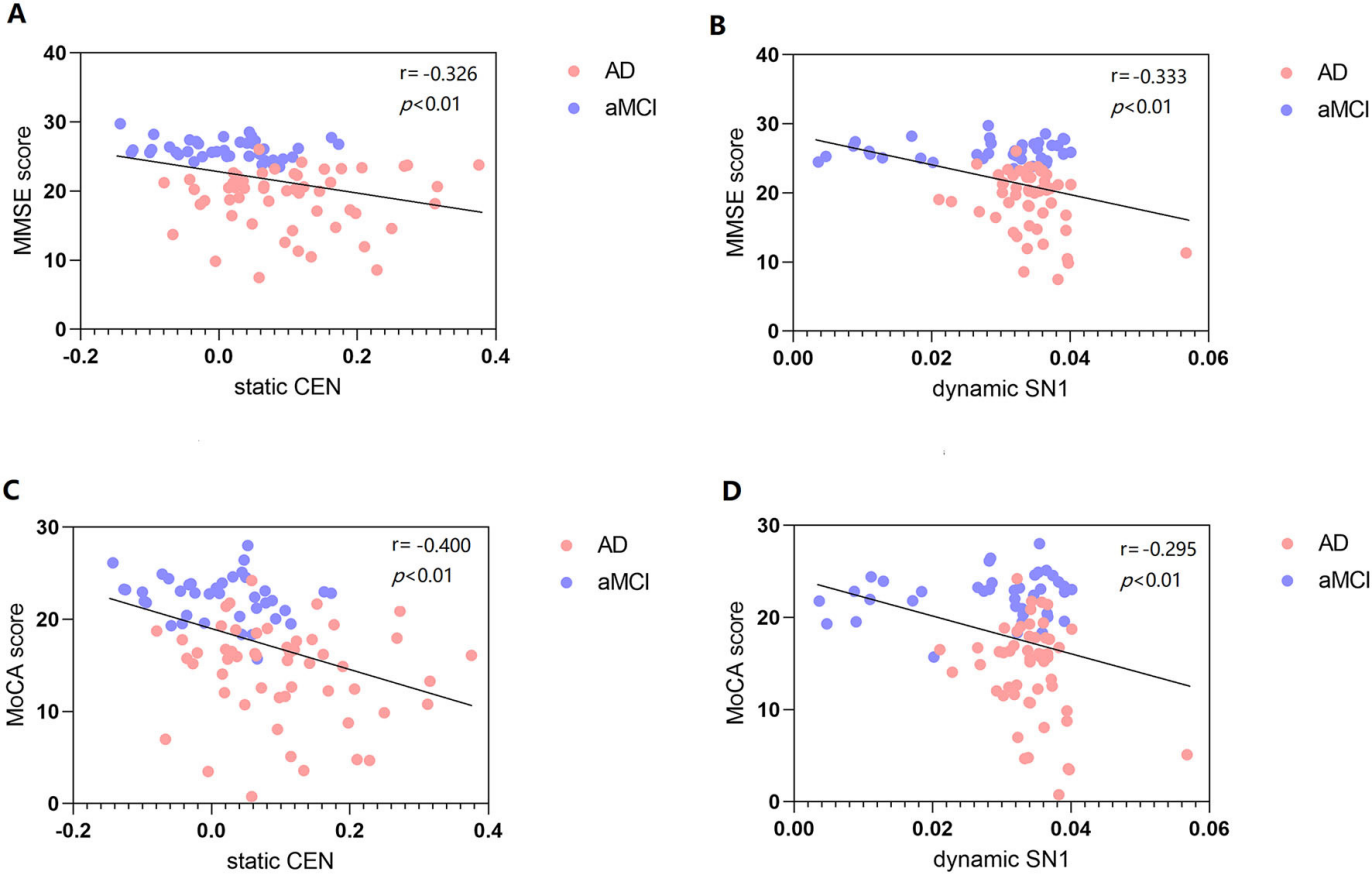
Correlation between MMSE score and static and dynamic FC **(A, B)**. Correlation between MoCA score and static and dynamic FC **(C, D)**. FC, functional connectivity; MMSE, mini-mental state examination; MoCA, Montreal cognitive assessment scale; CEN, central executive network; SN, salience network.

There was a significant negative correlation between sFC value of CEN (Left SPG) and MMSE and MoCA scores (*P* < 0.01, Bonferroni correction). There was a significant negative correlation between dFC value variability of SN1 (right Cerebelum_7b) and MMSE and MoCA scores (*P* < 0.01, Bonferroni correction) (Figure 5).

## Discussion

To our knowledge, the current study is one of the few that combines sFC and dFC and the triple network model to analyze AD and aMCI patients. The main findings of this study were that sFC and dFC variability within the three brain networks were altered to varying degrees. Furthermore, alterations in sFC within the CEN and dFC variability within the SN were significantly associated with cognitive scores in AD and aMCI patients. Most importantly, alterations in sFC and dFC variability, in combination with the triple network model, can be important biomarkers to improve the efficiency of diagnosing AD and aMCI.

### Static FC alteration of the triple networks

The human brain is connected through a complex network of functions that depend on each other to maintain cognitive function (39). Therefore, studying the static and dynamic states of brain functional network can better reflect the connectivity and activity of the resting state of the human brain, providing the basis for a more comprehensive understanding of the brain network in AD disease. In our previous study of 32 patients with AD, 26 patients with aMCI, and 58 NCs using rs-fMRI to detect directional FC in DMN, the AD group showed enhanced directional FC from the whole brain to the PCC, and weakened directional FC from the PCC to the whole brain within the DMN compared to the control group (34). Within the DMN network, the current study showed that there were significant differences in sFC values of right/left SFGmed. SFGmed is also a node of the DMN and is involved in its task processing. *Post hoc* tests showed that sFC values were significantly different between aMCI and NC, and the value of aMCI was elevated, suggesting the existence of compensation. Within the CEN network, there were significant differences in sFC values in left SPG. The functions of the CEN include: goal-directed cognition, inhibition, working memory, and task switch. The SPG is the somatosensory association cortex, which is related to spatial localization. *Post hoc* tests showed that sFC of SPG significantly decreased in aMCI compared to NC, suggesting that sFC is disrupted in the early stage of AD. As the disease progresses, sFC values increased, which is consistent with previous static FC studies (40), the increase in FC in AD stage may be a functional compensation after neurodegeneration.

### Dynamic FC alteration of the triple networks

Within the DMN network, the dFC variability in left IFGoperc and right SFGdor showed significant differences among the three groups. Brain area IFG plays an active role in emotion regulation. SFGdor is involved in various cognitive activities, mainly working memory, attention allocation, and cognitive manipulation execution. The SFGdor is also a key node of the DMN and the CEN. Within the SN network, there were significant differences in dFC variability of right Cerebelum_7b and left IFGoperc. SN plays an important role in identifying important or prominent information. A follow-up study showed that abnormal cerebellar FC is a more sensitive indicator of dysfunction in aMCI patients (41). An other study revealed that different rsFC patterns in cognitive-related sub-regions of the cerebellum (42). Cerebellar lesions may be part of the pathogenesis of aMCI, and more studies are needed to confirm the role of the cerebellum in AD spectrum disorders. Variability of dFC in the present study represents network flexibility. A larger CV indicates a more flexible network, while a smaller CV indicates a more stable network. From the results, the variability of dFC in DMN and SN2 was highest in NC, which indicates that the dynamic DMN and SN2 of NC group has higher flexibility. On the contrary, for dynamic SN1, we observed a similar pattern to static CEN, that is, compared with AD and NC, aMCI has the smallest dFC variability, which indicates that the dynamic SN1 of aMCI group has higher stability.

While most previous seed-based rs-fMRI studies have focused on the DMN, our study simultaneously included both DMN, SN, and CEN. We can speculate that AD and aMCI patients have both common and unique disruptions in the triple network. These three networks are directly or indirectly involved in cognitive tasks in the brain. Disruption of any of the three networks leads to abnormal internal psychological events and goal-related stimuli (43). Dynamic FC can capture repeated FC patterns that occur spontaneously and provide details such as FC strength or spatial dynamic properties that are averaged out in static brain network analysis (44). However, the changes in sFC and dFC within the triple network in AD and aMCI patients are not consistent. Other studies have found that SCD and aMCI groups have altered dFC variability in all three networks compared with healthy controls (17). However, the corrected test method they used in the ANOVA was not very rigorous, and they did not include static FC in the study. Although we did not find brain regions with altered sFC in the SN and altered dFC in the CEN, which may be related to the relatively strict GRF correction we adopted or the small sample size we included. In short, sFC combined with dFC provides a new entry point for further exploring the dynamic and static changes of neurons in the brain and revealing the pathogenesis of AD.

### Correlation between mental cognition scales and FC

There was a significant negative relevance between FC values (sFC values of CEN and dFC variability of SN1) and MMSE and MoCA scores. Among them, we found a negative correlation between dynamic SN1 and cognition, which indicates that smaller variability (higher stability) is more beneficial to cognition. However, we did not find a correlation between static and dynamic FC of DMN and cognition. Binnewijzend et al. suggested that DMN FC changes are associated with cognitive decline (45). A recent study also reported a correlation between intrinsic FC and cognitive function, which found that stronger FC between the PCC and the medial prefrontal lobe was associated with better performance of working memory (46). The connectivity changes in the dFC state reflect the complex neuroregulatory mechanism in the brain and are closely related to behavior.

### Limitations

There are still some limitations to our study. First, we lack pathological biomarkers and genetic data. Secondly, using a large number of nuisances regressors in rs-fMRI may lead to overfitting the data, resulting in removing the signal of interest. Finally, our sample size was small. The correlation analysis was performed only in the AD+aMCI groups, not separately among the three groups. In the future, we will recruit more subjects, and conduct follow-up, while collecting AD pathological and genetic data to further confirm the results of this study.

## Conclusion

In summary, the current study is one of the few to combine sFC and dFC and triple network models to analyze patients with AD and aMCI. Our study found that the variability of sFC and dFC within the triple network model was altered to varying degrees in AD and aMCI. Our study also demonstrated that alterations in sFC and dFC of the triple network model were significantly associated with cognitive performance in AD and aMCI patients. Therefore, it suggests that sFC, dFC combined with the triple network model can be considered as neuroimaging biomarkers for AD and aMCI diagnosis.

## Data availability statement

The raw data supporting the conclusions of this article will be made available by the authors, without undue reservation.

## Ethics statement

The studies involving humans were approved by the Ethics Committee of Zhejiang Provincial People's Hospital (No. 2012KY002). The studies were conducted in accordance with the local legislation and institutional requirements. The participants provided their written informed consent to participate in this study.

## Author contributions

QF: Writing – original draft, Writing – review & editing, Formal analysis. LW: Formal analysis, Methodology, Writing – original draft. XT: Data curation, Methodology, Writing – original draft. HH: Data curation, Software, Writing – original draft. XG: Funding acquisition, Validation, Writing – review & editing. ZL: Conceptualization, Supervision, Writing – review & editing. ZD: Conceptualization, Funding acquisition, Writing – review & editing, Supervision.
