# Supplementary material for: Static and dynamic functional connectivity combined with the triple network model in amnestic mild cognitive impairment and Alzheimer's disease
Source: Front Neurol. 2023 Nov 17;14:1284227. doi: 10.3389/fneur.2023.1284227 (PMC10723161; doi:10.3389/fneur.2023.1284227)
Supplement: Supplementary file 1 [file Data_Sheet_1.docx]

Static and dynamic functional connectivity combined with the triple network model in amnestic mild cognitive impairment and Alzheimer's disease


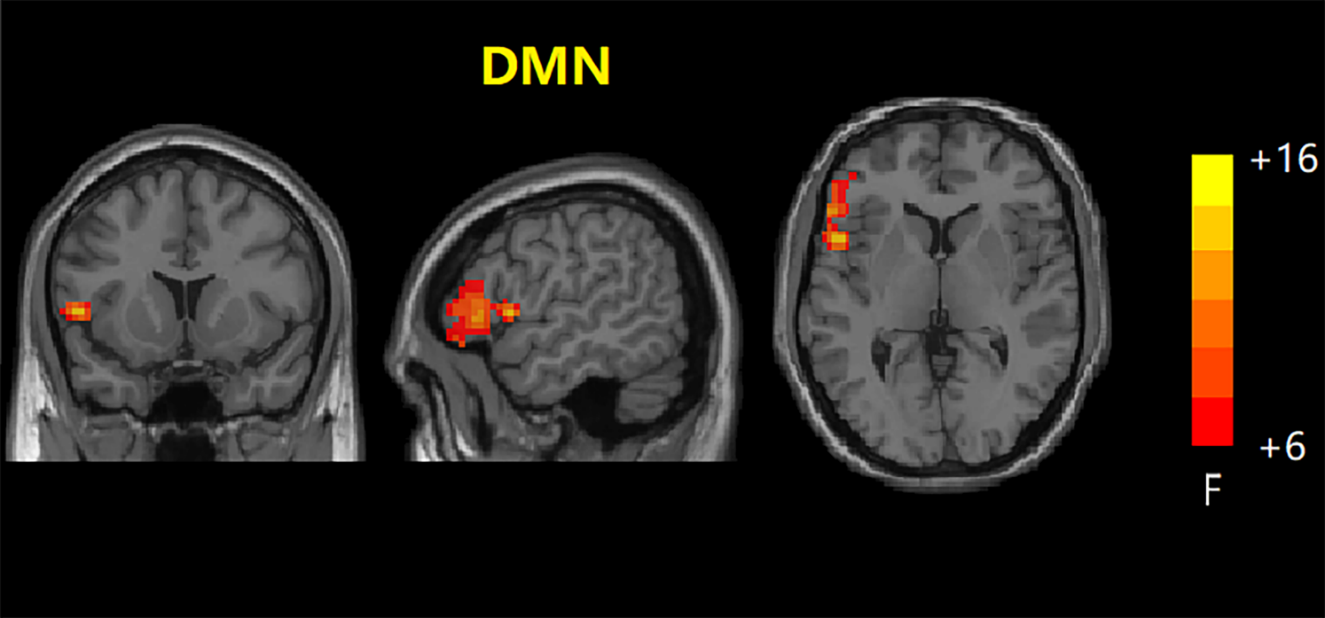


**FIGURE 1 |** Dynamic FC results among the three groups using 60 TR sliding window length and a 2 TR step. Brain regions with significant differences in dFC variability in DMN network. Abbreviations: DMN, default mode network.


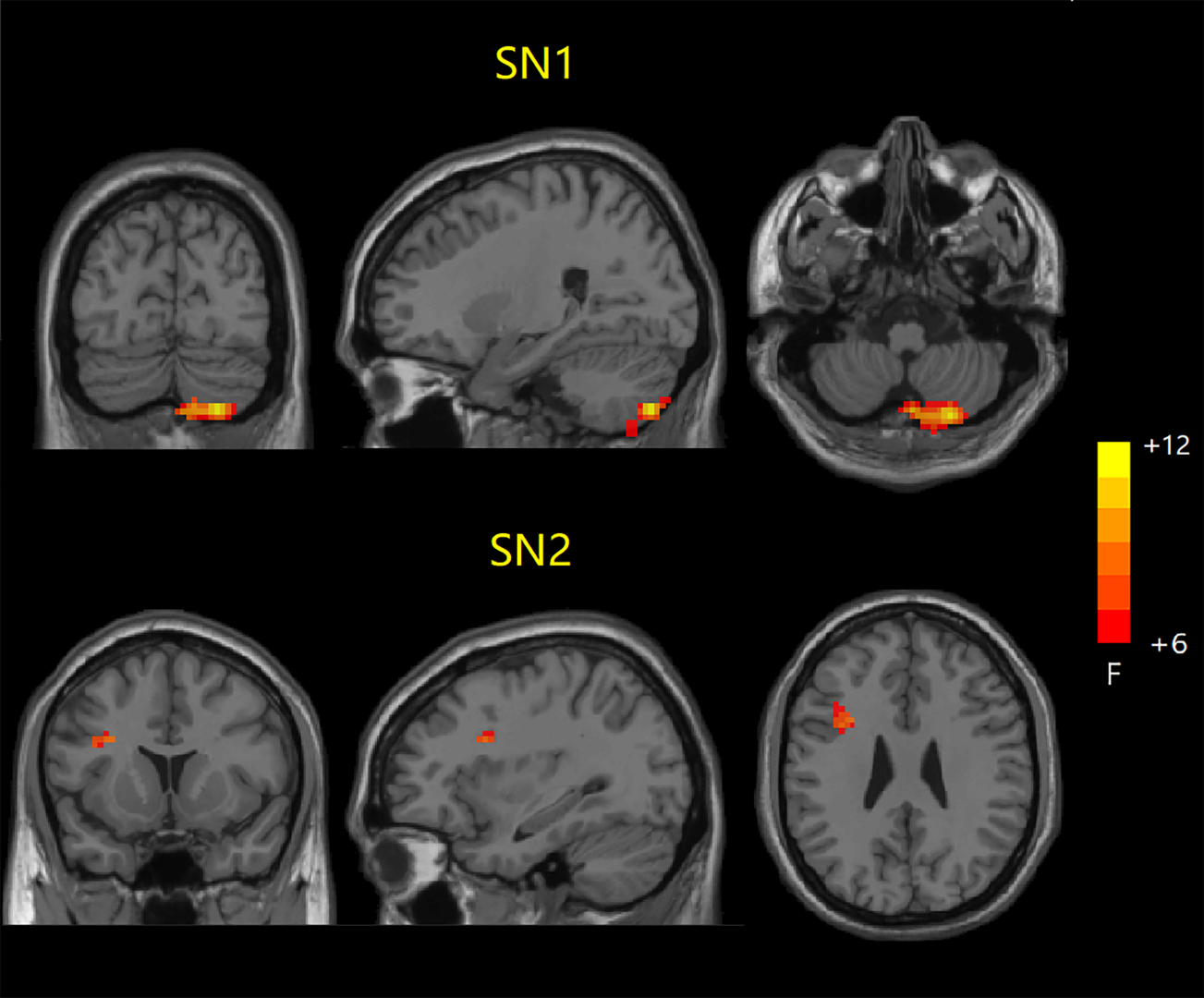


**FIGURE 2 |** Dynamic FC results among the three groups using 60 TR sliding window length and a 2 TR step length. Brain regions with significant differences in dFC variability in SN network. Abbreviations: SN, salience network.


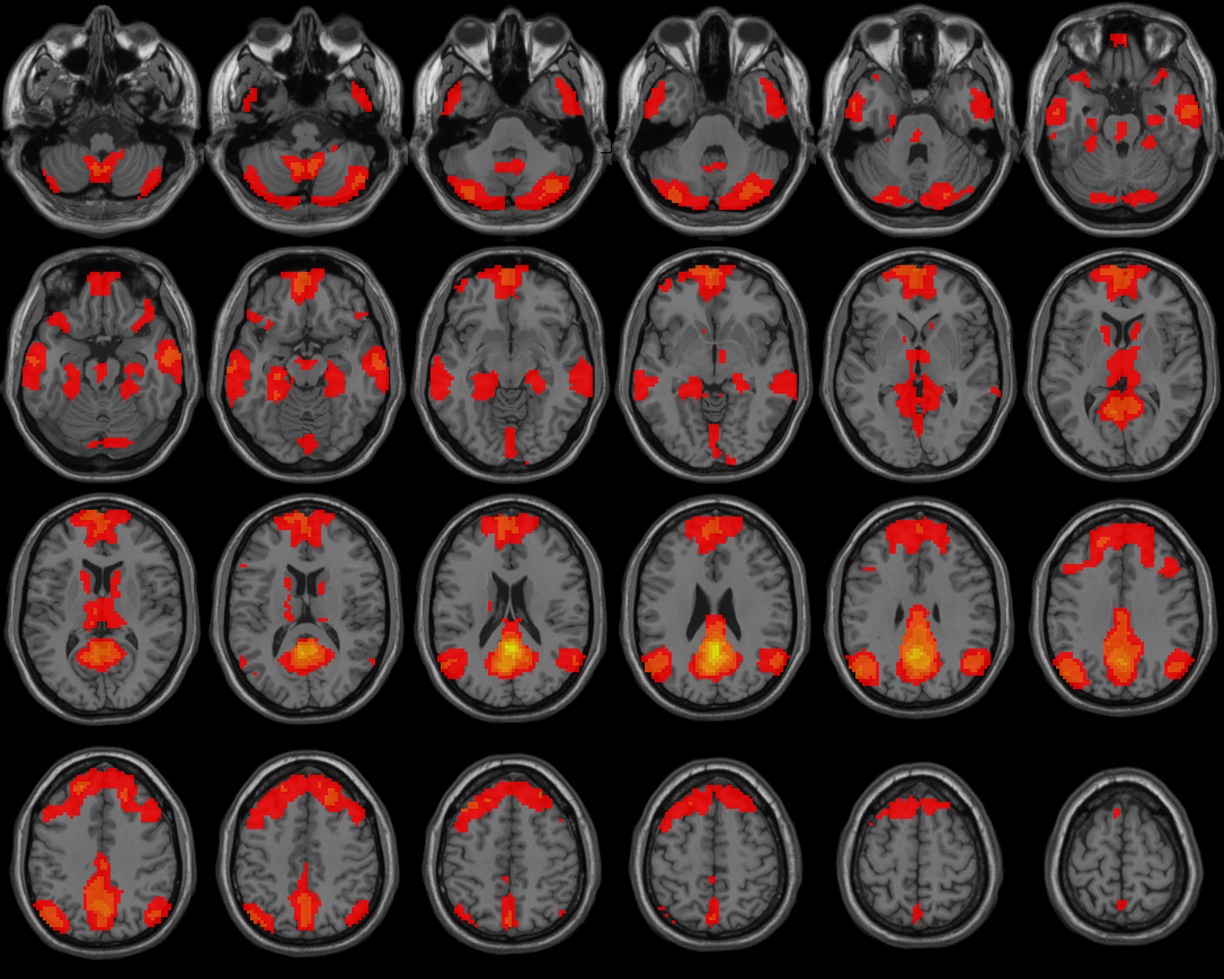


**FIGURE 3 |** Typical DMN pattern in AD group.


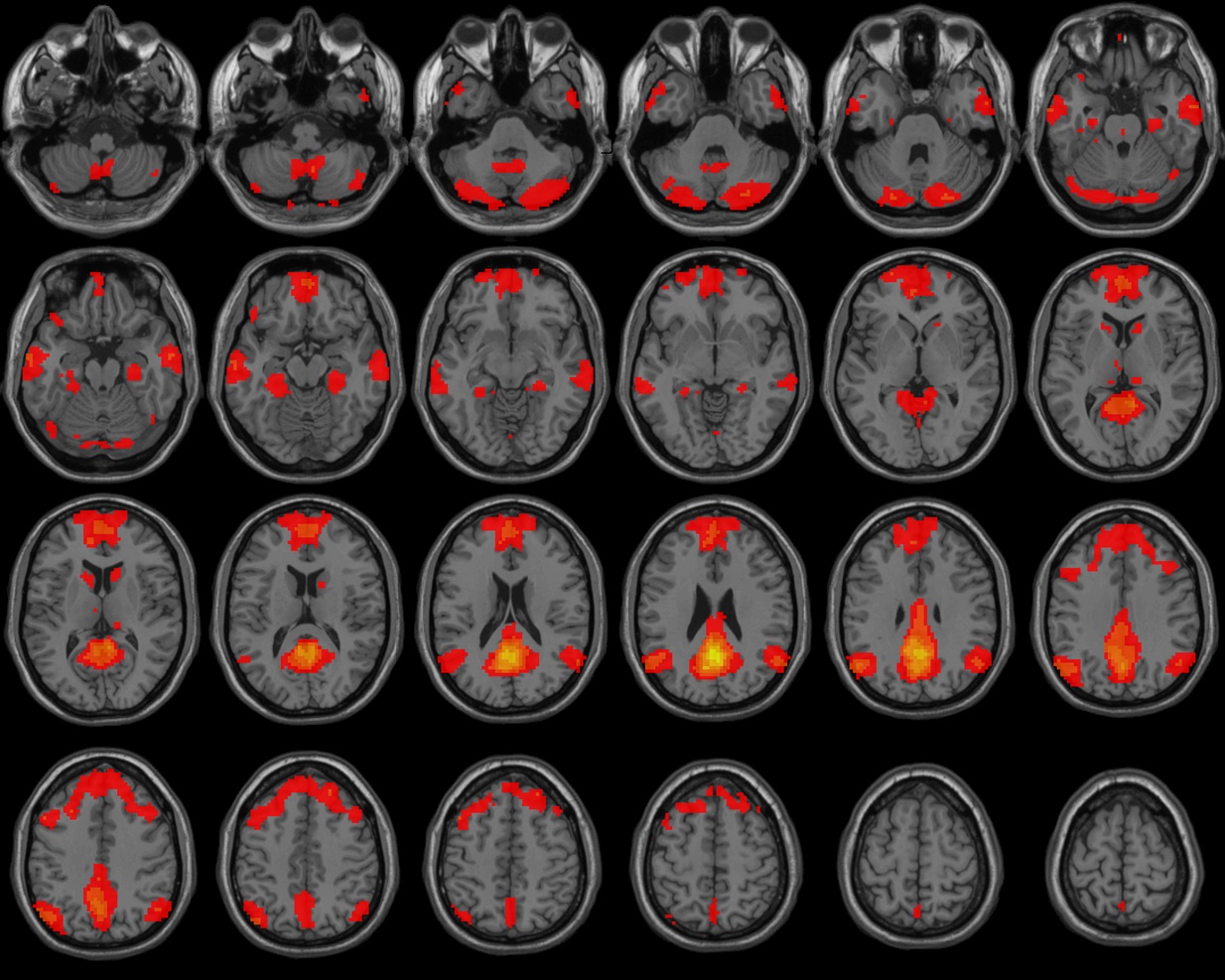


**FIGURE 4 |** Typical DMN pattern in aMCI group.


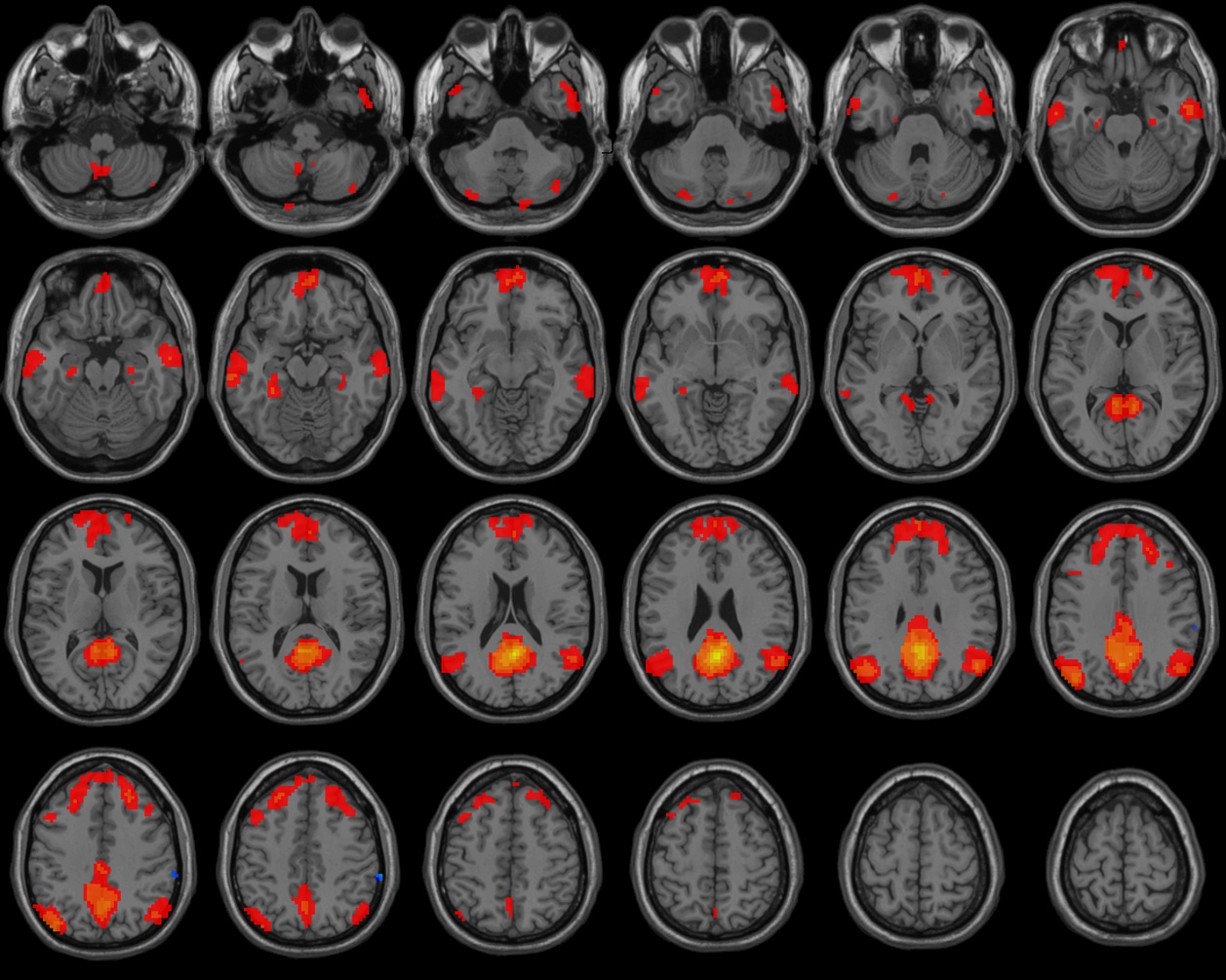


**FIGURE 5 |** Typical DMN pattern in NC group.


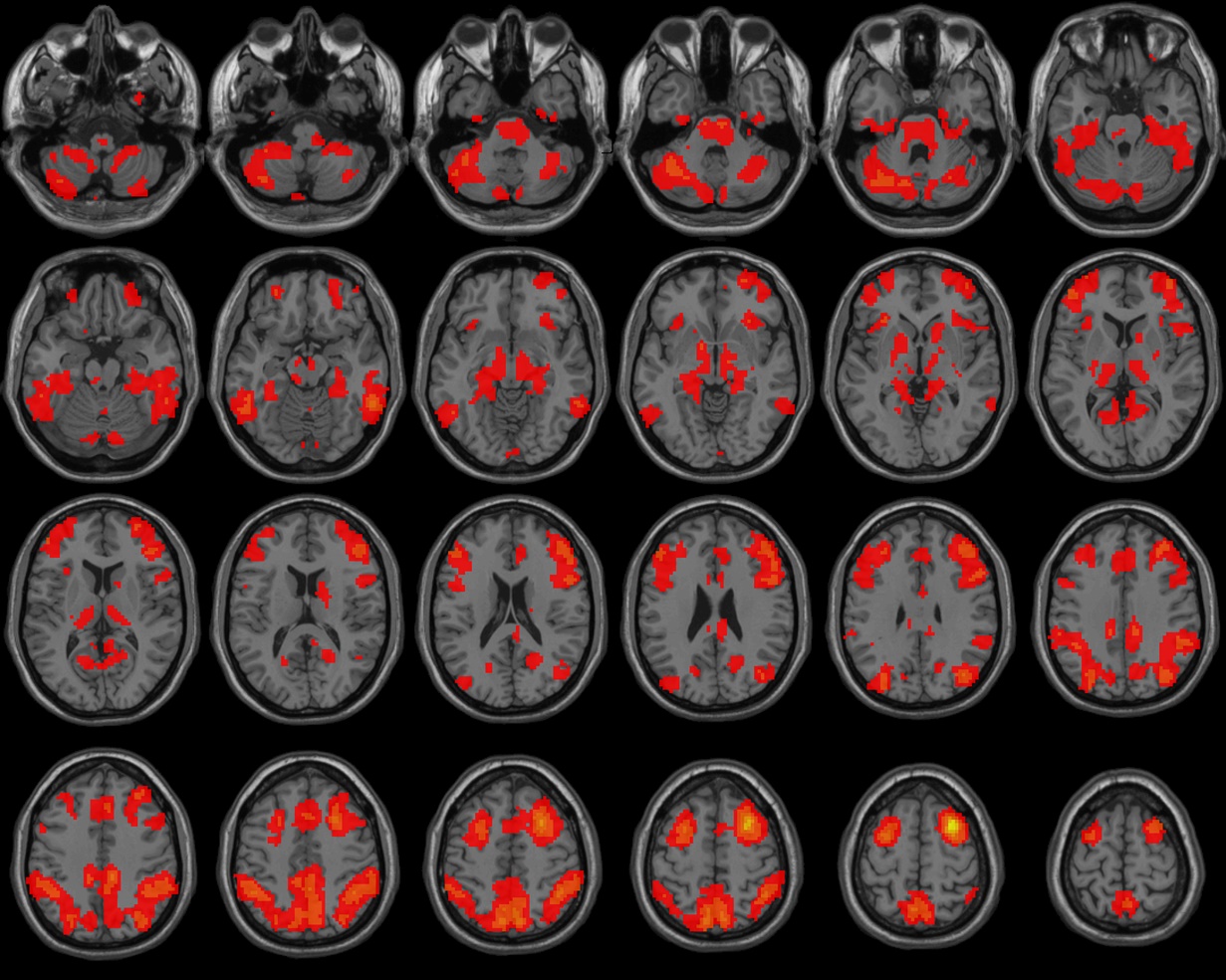


**FIGURE 6 |** Typical CEN pattern in AD group.


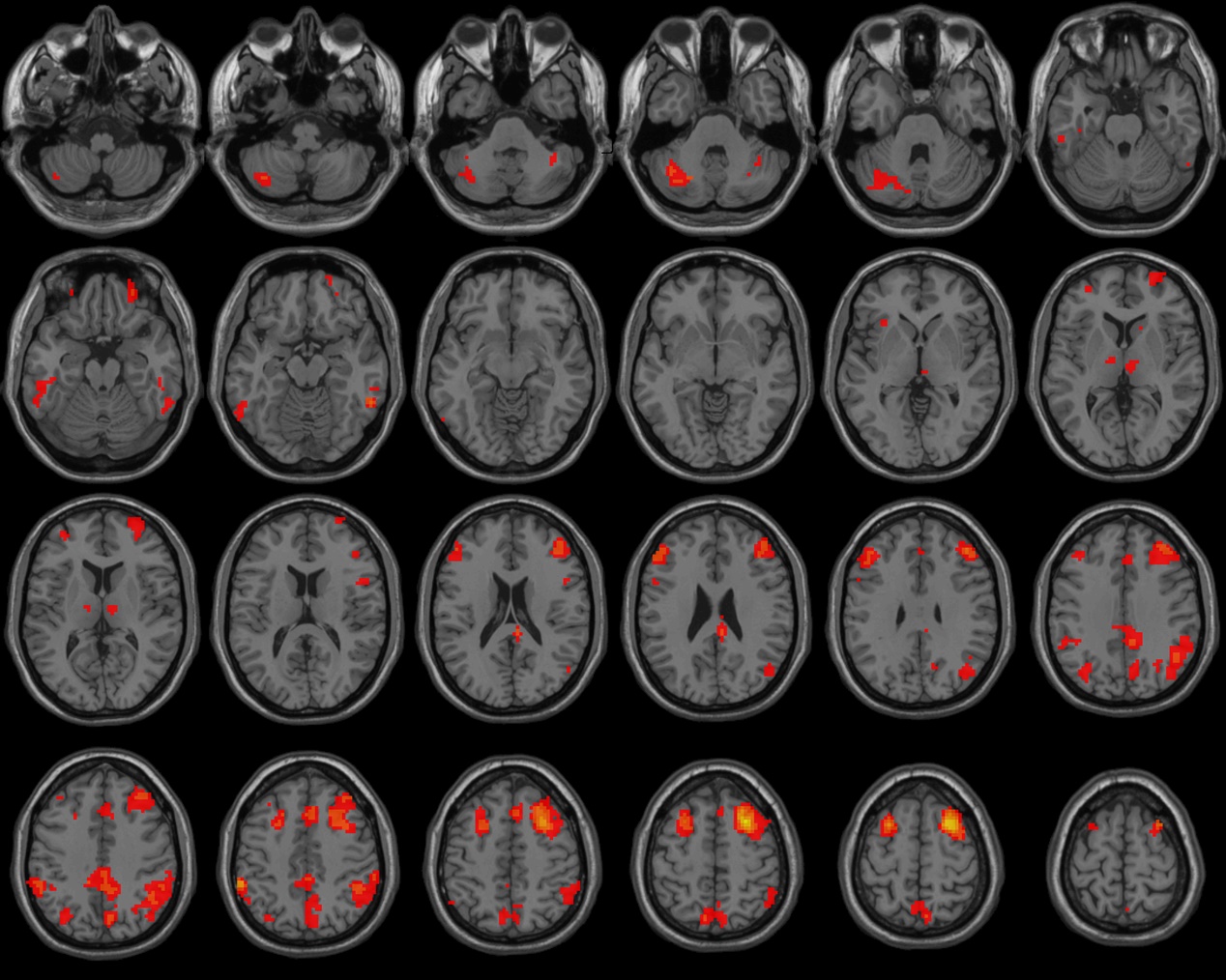


**FIGURE 7 |** Typical CEN pattern in aMCI group.


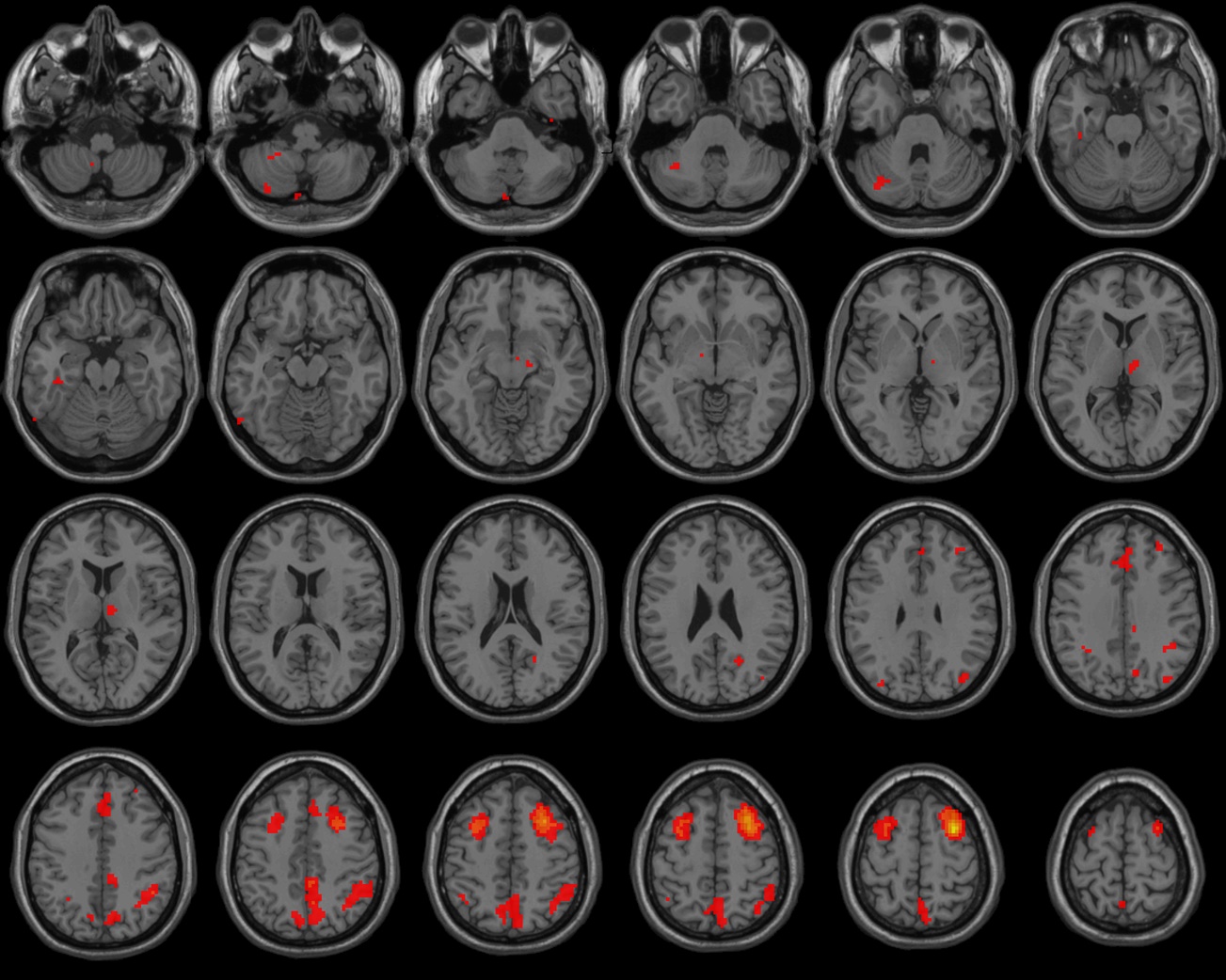


**FIGURE 8 |** Typical CEN pattern in NC group.


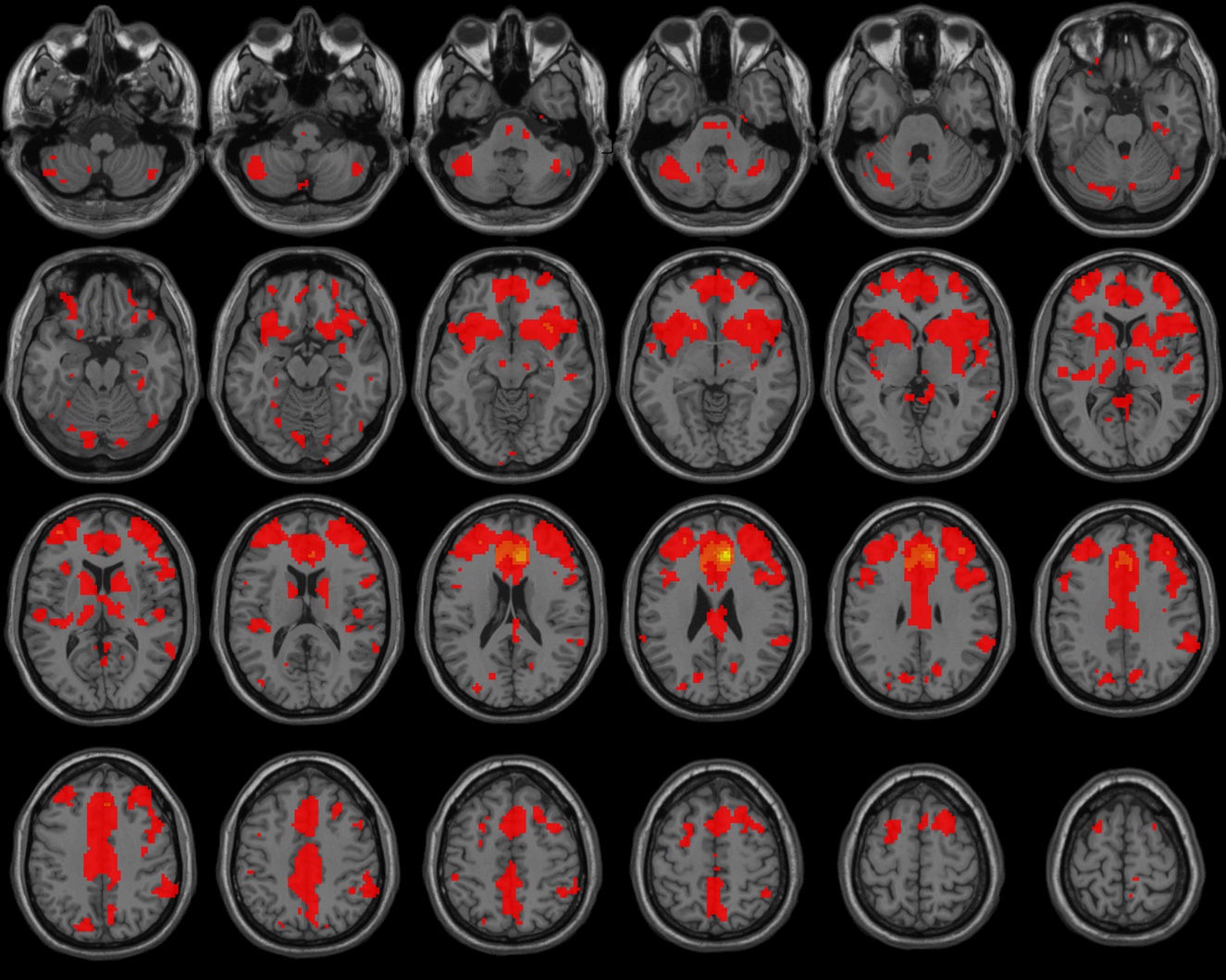


**FIGURE 9 |** Typical SN pattern in AD group.


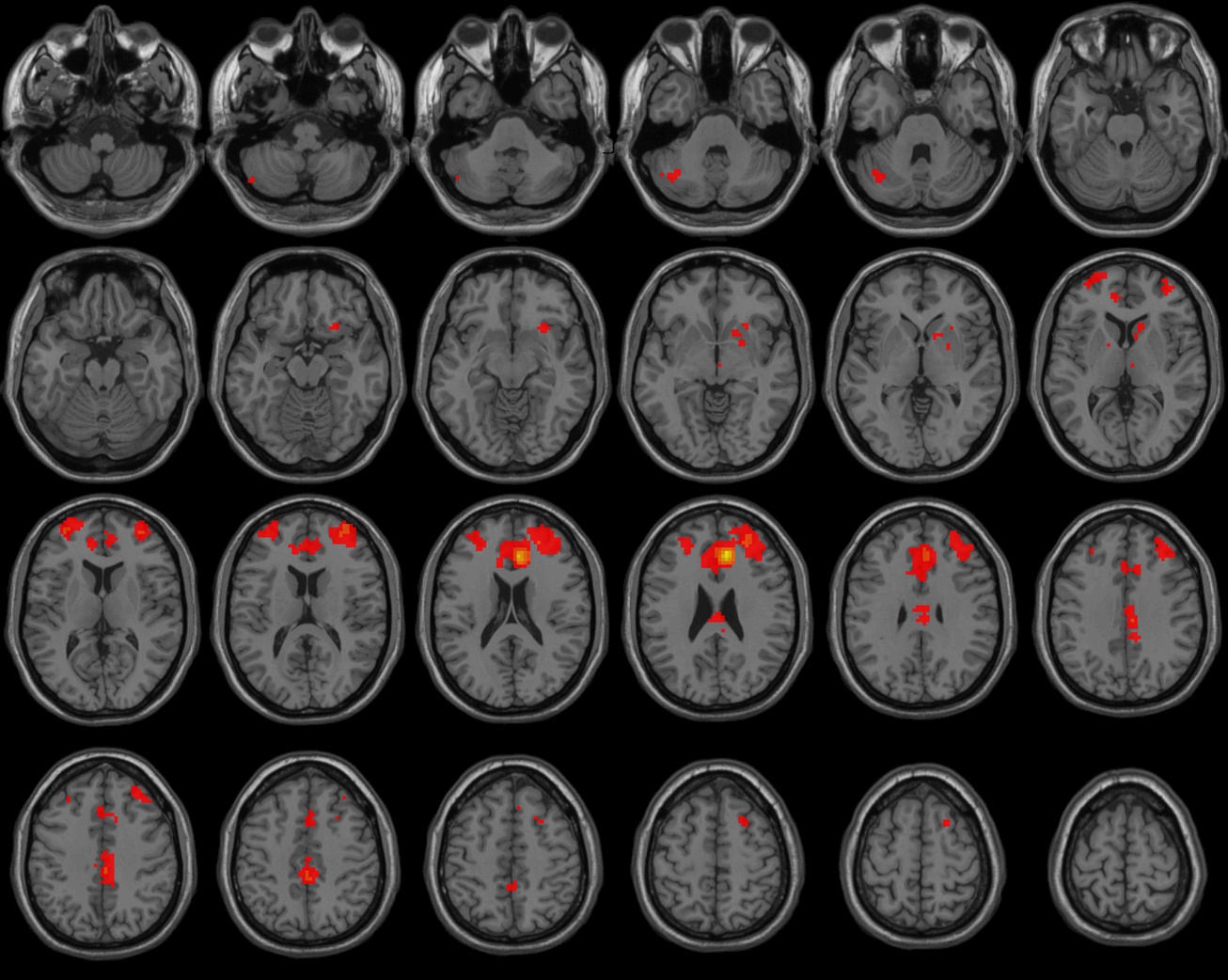


**FIGURE 10 |** Typical SN pattern in aMCI group.


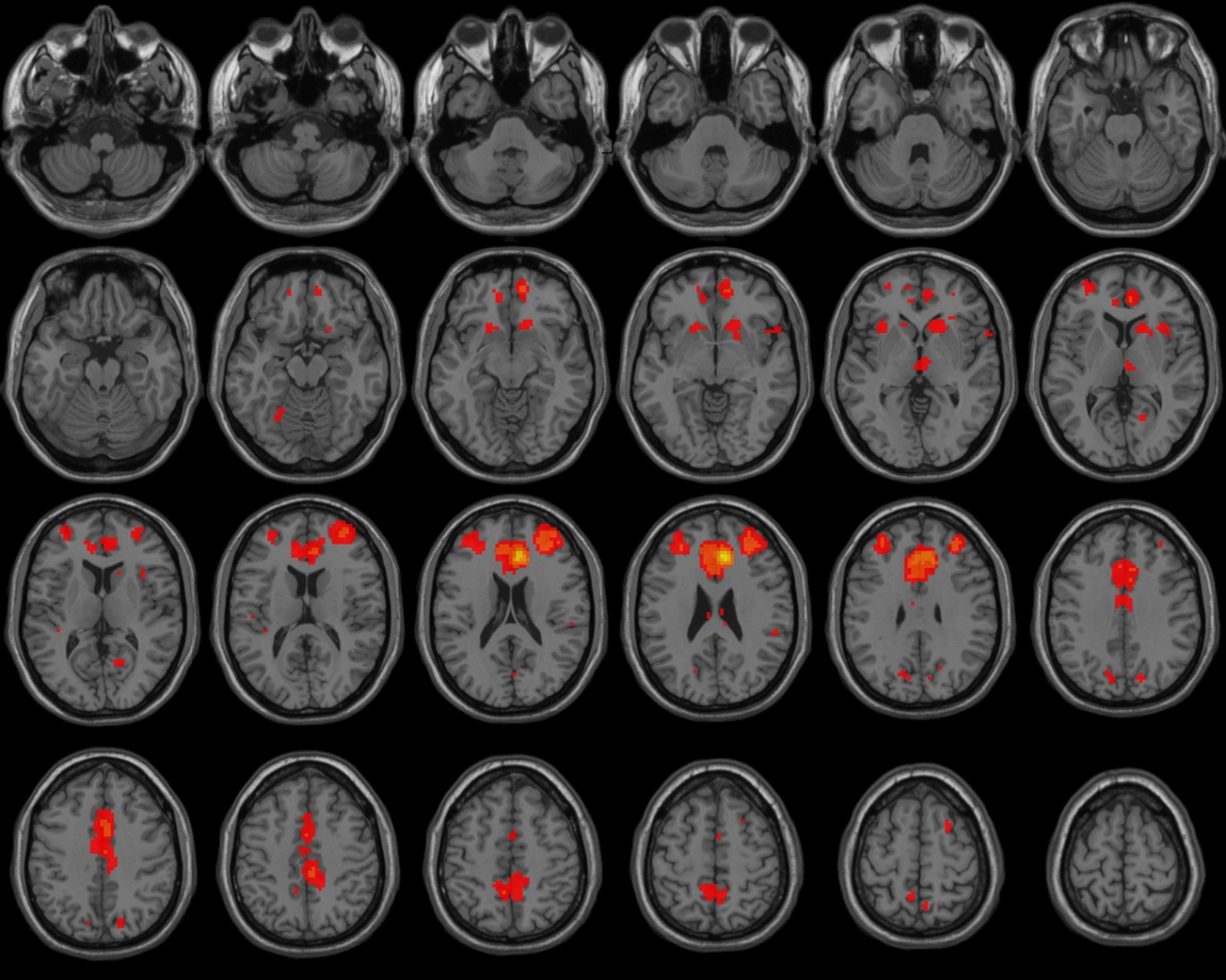


**FIGURE 11 |** Typical SN pattern in NC group.

**TABLE 1 | Correlation between mental cognition scales and static and dynamic FC.**

| Network metrics | MMSE | | | | MoCA | | | |
| --- | --- | --- | --- | --- | --- | --- | --- | --- |
|  | AD | aMCI | NC | AD+aMCI | AD | aMCI | NC | AD+aMCI |
| Static DMN | r = -0.054  *P* = 0.711 | r = 0.005  *P* =0.977 | r = 0.013  *P* =0.938 | r = 0.202  *P* =0.057 | r = -0.048  *P* = 0.741 | r = -0.312  *P* =0.064 | r = 0.061  *P* =0.724 | r = 0.163  *P* =0.127 |
| Static CEN | r = 0.024  *P* = 0.872 | r = -0.100  *P* =0.563 | r = 0.102  *P* =0.554 | **r = -0.329**  ***P* =0.002** | r = -0.121  *P* = 0.407 | r = -0.076  *P* =0.659 | r = 0.031  *P* =0.857 | **r = 0.400**  ***P* <0.001** |
| Dynamic DMN1 | r = -0.154  *P* = 0.291 | r = 0.049  *P* =0.775 | r = -0.171  *P* =0.318 | r = 0.216  *P* =0.042 | r = -0.154  *P* = 0.289 | r = 0.099  *P* =0.565 | r = -0.157  *P* =0.361 | r = 0.203  *P* =0.056 |
| Dynamic DMN2 | r = -0.238  *P* = 0.100 | r = 0.031  *P* =0.857 | r = -0.211  *P* =0.216 | r = 0.241  *P* =0.023 | r = -0.234  *P* = 0.105 | r = -0.063  *P* =0.715 | r = 0.100  *P* =0.562 | r = 0.198  *P* =0.063 |
| Dynamic SN1 | r = -0.273  *P* = 0.057 | r = 0.262  *P* =0.122 | r = -0.074  *P* =0.667 | **r = -0.333**  ***P* =0.001** | r = -0.279  *P* = 0.052 | r = 0.228  *P* =0.181 | r = -0.034  *P* =0.844 | **r = -0.295**  ***P* =0.005** |
| Dynamic SN2 | r = -0.153  *P* = 0.295 | r = 0.063  *P* =0.714 | r = -0.336  *P* =0.045 | r = 0.108  *P* =0.315 | r = -0.167  *P* = 0.253 | r = 0.051  *P* =0.769 | r = 0.516  *P* =0.001 | r = 0.078  *P* =0.470 |
